# SAA suppresses α-PD-1 induced anti-tumor immunity by driving T_H_2 polarization in lung adenocarcinoma

**DOI:** 10.1038/s41419-023-06198-w

**Published:** 2023-11-04

**Authors:** Xin Wang, Shaodi Wen, Xiaoyue Du, Yihan Zhang, Xiao Yang, Renrui Zou, Bing Feng, Xiao Fu, Feng Jiang, Guoren Zhou, Zi Liu, Wei Zhu, Rong Ma, Jifeng Feng, Bo Shen

**Affiliations:** 1 Department of Oncology, The Affiliated Cancer Hospital of Nanjing Medical University, Jiangsu Cancer Hospital, Jiangsu Institute of Cancer Research, Nanjing, China; 2 Department of Clinical Laboratory, Jiangsu Cancer Hospital, The Affiliated Cancer Hospital of Nanjing Medical University, Jiangsu Institute of Cancer Research, Nanjing, China; 3 Department of General Surgery, Nanjing Drum Tower Hospital, Nanjing, China; 4 Clinical College of Traditional Chinese and Western Medicine, Nanjing University of Chinese Medicine, Nanjing, China; 5 Department of Thoracic Surgery, Jiangsu Cancer Hospital, The Affiliated Cancer Hospital of Nanjing Medical University, Jiangsu Institute of Cancer Research, Nanjing, China; 6 Nanjing Advanced Analysis Tech. (NAAT) Co., LTD, Nanjing, China; 7 School of Medicine, Jiangsu University, Zhenjiang, China; 8 Research Center for Clinical Oncology, Jiangsu Cancer Hospital & Jiangsu Institute of Cancer Research & The Affiliated Cancer Hospital of Nanjing Medical University, Nanjing, China

**Keywords:** Non-small-cell lung cancer, Translational research

## Abstract

Cancer stem cells (CSCs) are believed to be crucial in the initiation, progression, and recurrence of cancer. CSCs are also known to be more resistant to cancer treatments. However, the interaction between CSCs and the immune microenvironment is complex and not fully understood. In current study we used single cell RNA sequence (scRNA-Seq, public dataset) technology to identify the characteristic of CSCs. We found that the lung adenocarcinoma cancer stem population is highly inflammatory and remodels the tumor microenvironment by secreting inflammatory factors, specifically the acute phase protein serum amyloid A (SAA). Next, we developed an ex-vivo autologous patient-derived organoids (PDOs) and peripheral blood mononuclear cells (PBMCs) co-culture model to evaluate the immune biological impact of SAA. We found that SAA not only promotes chemoresistance by inducing cancer stem transformation, but also restricts anti-tumor immunity and promotes tumor fibrosis by driving type 2 immunity, and α-SAA neutralization antibody could restrict treatment resistant and tumor fibrosis. Mechanically, we found that the malignant phenotype induced by SAA is dependent on P2X7 receptor. Our data indicate that cancer stem cells secreted SAA have significant biological impact to promote treatment resistant and tumor fibrosis by driving cancer stemness transformation and type 2 immunity polarization via P2X7 receptor. Notably, α-SAA neutralization antibody shows therapeutic potential by restricting these malignant phenotypes.

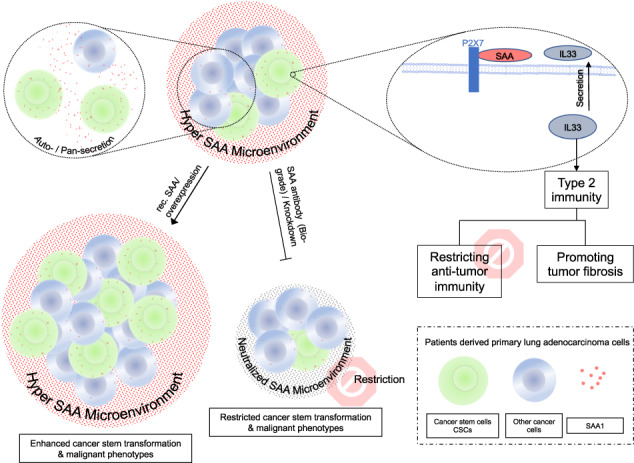

## Introduction

A large proportion of lung adenocarcinoma patients have lost the opportunity for surgery when they are first diagnosed and can only rely on non-surgical treatments such as chemotherapy, target therapy and newly developed immunotherapy [1]. However, treatment resistance has been a persistent issue that has plagued clinical treatment for many years [2].

Intratumor heterogeneity is indeed one of the major reasons for treatment resistance in cancer. Tumors are composed of different types of cells with different genetic, epigenetic and phenotypic background / properties, which can make them respond differently to different treatments [3, 4]. Cancer stem cells (CSCs) are a subpopulation of tumor cells that possess stem cell-like properties and are responsible for tumor initiation, maintenance, and resistance to therapy [5–7].

In our previous study, we identified a subpopulation of highly primitive cancer stem cells in primary lung adenocarcinoma (LUAD) using flow cytometry. These cells were characterized by CD133^+^/CD44^+^/CD24^-^ and displayed resistance to chemotherapy [8]. In current study, we further identified that the CD133^+^ cancer stem cell population is also highly inflammatory and secretes many inflammation factors. Among them, we identified that Serum amyloid A (SAA) in the tumor microenvironment is primarily secreted by the cancer stem cells. SAA is a type of acute-phase protein that is produced in response to inflammation and involved in regulation of immune responses, tissue repair, and tumor progression [9]. SAA has been shown to promote tumor growth, angiogenesis, and metastasis by modulating the tumor microenvironment. High levels of SAA in the blood or tumor tissue have been associated with poor prognosis in various types of cancers, including lung cancer [10–13].

Smole et al. reported that SAA is a soluble pattern recognition receptor that drives type 2 immunity [14], indicating CSCs secreted SAA may also involve in the regulation of anti-cancer immunity. Type 1 and Type 2 immunity are two major types of immune responses, type 1 immunity is characterized by the activation of T helper 1 (T_H_1) cells and the production of cytokines such as interferon-gamma (IFN-γ), which are important for the elimination of intracellular pathogens and tumor cells. Type 2 immunity, on the other hand, is characterized by the activation of T helper 2 (T_H_2) cells and the production of cytokines such as interleukin-4 (IL-4), which are important for the elimination of extracellular parasites and allergens [15–17]. In terms of anti-cancer immunity, type 1 immunity is generally considered to be more effective than type 2 immunity, as it can activate cytotoxic T lymphocytes (CTLs) to recognize and kill tumor cells [18–20]. Type 2 immunity is also strongly associated with wound healing and fibrosis. Excessive type 2 immunity, particularly the associated tissue fibrosis, can lead to an immunosuppressive tumor microenvironment and promote tumor growth and metastasis [15, 21]. Thus, we further explored the impact of SAA on the balance between type 1 and type 2 immunity.

In the current study, we identified that SAA is primarily secreted by the cancer stem cell population. To investigate the biological immune impact of SAA, we introduced recombinant SAA and α-SAA neutralization antibody into an ex-vivo tumor organoids-PBMCs (peripheral blood mononuclear cell) model. We observed that SAA promotes cancer stemness transformation, while α-SAA significantly restricts the expansion of cancer stem cells. Moreover, we found that SAA signaling suppresses anti-tumor immunity and promotes tumor fibrosis by driving type 2 immunity, and α-SAA neutralization antibody may have therapeutic potential.

## Results

### Cancer stem population is highly inflammatory and secretes SAA

**Fig. 1:**
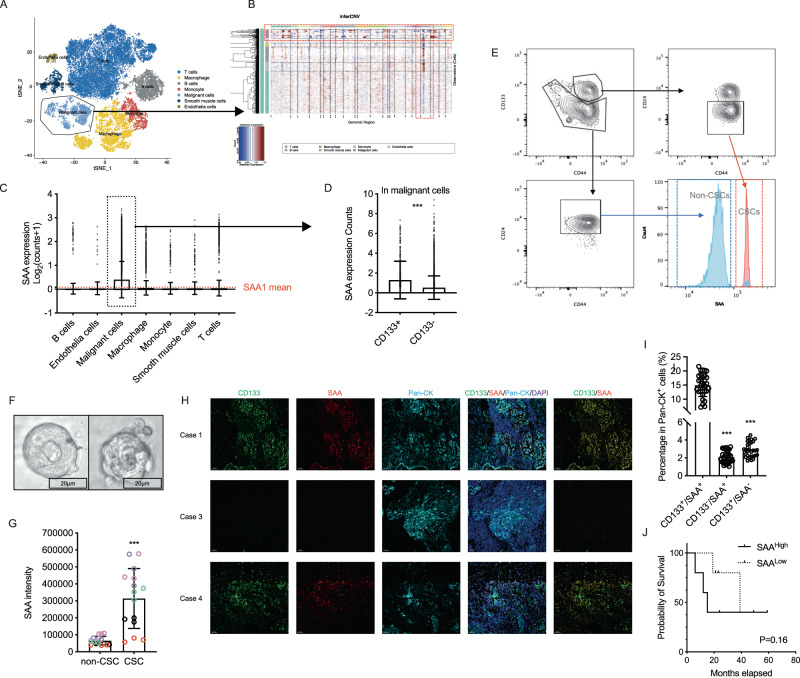
Cancer stem cells are highly inflammatory and secret SAA. **A**, **B** The GSE131907 were introduced for single cell mRNA and copy number variation analysis in malignant cells (for more details about scRNA-Seq analysis in Fig. S1). **C** Expression of SAA in different cell subpopulations (mean with SD, one way ANOVA with Post Hoc analysis). **D** Expression of SAA between CD133^+^ or CD133^-^ malignant populations (mean with SD, *t*-test). **E**–**G** The LUAD tumor organoids were established, and the intracellular SAA level were measured by flow cytometry in CD133^+^ and CD133^-^ cancer cell populations (mean with SD, *t*-test). **H** Ten lung cancer FFPE samples were enrolled for immunofluorescence imaging, the SAA, CD133, Pan-CK and DAPI were stained. **I** The percentage of different subpopulation were counted (mean with SD, one way ANOVA with Post Hoc analysis). **J** The PFS KM plot for enrolled lung cancer patients (Gehan–Breslow–Wilcoxon test).

Deep profiling of cellular heterogeneity requires multiplex single cell based technology, such as scRNA-Seq, flow cytometry or Immunofluorescence staining (with quantitative analysis). We first utilized a robust scRNA dataset comprising of 11 tumor samples from the publicly available GSE131907 dataset [22]. To ensure the high quality of the data, we performed a comprehensive quality control analysis, including an assessment of the cell count depth, the number of genes detected, and the number of mitochondrial genes (Fig. S1-1A-F) [23]. Ultimately, we identified 9 primary lung adenocarcinoma samples that met our stringent quality control standards. Next, we employed the Seurat package to cluster the cells using the t-distributed stochastic neighbor embedding (tSNE) algorithm, then we identified a malignant population with disordered copy number variation (Fig. 1A, B). Even though all the samples analyzed in our study were derived from LUAD tissue, we observed variation in cellular composition across these samples (Fig. S1G).

Next, we analyzed bulk samples from TCGA_LUAD dataset, the top 5 and bottom 5 CD133 (PROM1) expression samples were selected for GSEA analysis. Our results showed a significant correlation between CD133 and EMT (epithelial-mesenchymal transition) as well as inflammatory responses (Figs. S1–2A and C). This finding suggests that CD133 is a reliable stemness biomarker, and cancer stemness is linked to inflammatory processes. We also observed the high expression of the acute phase protein SAA1/2 in the CD133^hi^ group (Fig. S1-2B). After that, we measured the distribution of SAA between different major subpopulations in scRNA data, we found that SAA was mainly expressed in malignant epithelial cells, and CD133^+^ malignant cells express more SAA which indicating this primitive cancer stem population was more inflammatory (Fig. 1C, D).

For evaluating the distribution of SAA protein in different LUAD subpopulation we used patient derived organoids (Fig. 1E, F), in tumor organoids we first identified Pan-CK^+^/CD133^+^/CD44^+^/CD24^-^ population as primitive cancer stem population, and in intracellular staining we observed significantly higher level of SAA signal in cancer stem population (Fig. 1G). Furthermore, in clinical samples, we observed that most CD133^+^ lung cancer malignant cells express high levels of SAA (*n* = 10, Fig. 1H, I). However, due to the limited sample size, we were unable to establish a correlation between SAA expression and patient survival (Fig. 1J). In summary we found that, the primitive cancer stem population have inflammatory phenotype and express higher level of SAA.

### SAA promotes chemo-resistant by driving stemness transformation and cell quiescence

Elevated levels of SAA have been found in several types of cancer, and there is evidence suggest that SAA can promote tumor growth and metastasis [10, 24], we further explored the biological impact of SAA in ex-vivo organoids model.

**Fig. 2:**
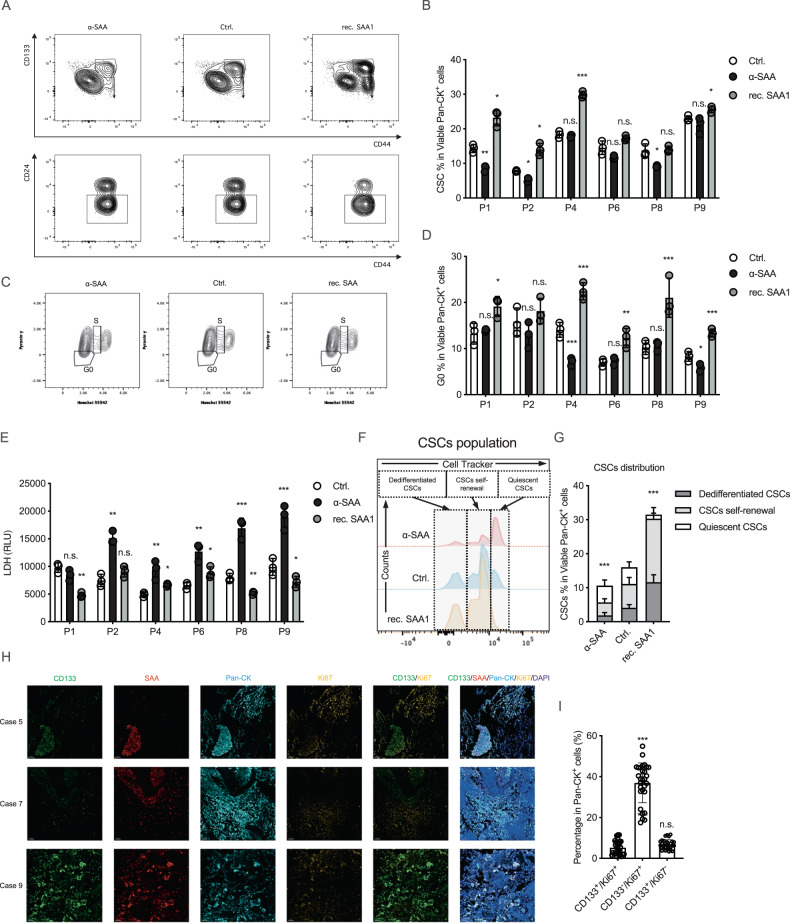
SAA promotes chemo-resistant by driving stemness transformation and cell quiescence. **A**, **B** α-SAA neutralization antibody and rec. SAA1 protein were introduced in the tumor organoids model, and the CD133^+^/CD44^+^/CD24^-^ cancer stem population were immunophenotyped by flow cytometry in 6 organoids samples (mean with SD, one way ANOVA with Post Hoc analysis). **C**, **D** The Hoechst 33342 and Pyronin γ double negative population was identified as G0 (quiescent) population in malignant cells (mean with SD, one way ANOVA with Post Hoc analysis). **E** 5 μM cDDP was introduced in organoids model, and the supernatant were collected for LDH measurement (cytotoxic assay) (mean with SD, one way ANOVA with Post Hoc analysis). **F**, **G** The CSCs cells were sorted by flow cytometry and tagged with CellTracker, after that the tagged CSCs cells were mix with other cancer cells, 7 days later the cells were immunophenotyped by flow cytometry and the CellTracker intensity were measured in CSCs population (mean with SD, one way ANOVA with Post Hoc analysis). **H** Ten lung cancer FFPE samples were enrolled for immunofluorescence imaging, the SAA, CD133, Pan-CK, Ki-67 and DAPI were stained. **I** The percentage of different subpopulation were counted (mean with SD, one way ANOVA with Post Hoc analysis).

We introduced SAA neutralization antibody (α-SAA, 500 μg/ml) and recombinant human SAA1 protein (rec. SAA1, 50 μg/ml) in organoids culture model, and the best effecting concentration of α-SAA and rec. SAA were determined by measuring their ability to induce or suppress stemness transformation in organoid sample in a serial dilution assay (Fig. S2A). We found that, neutralizing SAA limits the expansion of cancer stem population (3/6), while on the other side introducing rec. SAA in tumor microenvironment fertilizes the expansion of cancer stem population (3/6, Fig. 2A, B). Moreover, we found that SAA also promotes cell quiescence (Fig. 2C, D), we further introduced 5 µM cDDP (cisplatin) in organoids ex-vivo culture model, we found that neutralizing SAA suppresses chemoresistance potently (5/6), while rec. SAA promotes chemoresistance (5/6, Fig. 2E).

Next, to investigate whether SAA promotes stemness transformation by promoting the self-renewal of cancer stem population or by inducing dedifferentiation of other tumor cells, we sorted CD133^+^ cancer stem cell population and labeled them with CellTracker, subsequently, the tagged cancer stem cells were mixed again with other tumor cells and cultured in organoid medium with recombinant SAA1 or α-SAA for 5 days. We found that, SAA signaling promotes both CSCs self-renewal and non-CSCs dedifferentiation simultaneously, but SAA signaling primarily promotes stemness transformation by promoting the self-renewal of CSC population (Fig. 2F, G). For clinical validation, we enrolled ten lung cancer FFPE samples for multiplex imaging of SAA, CD133, and Ki67. Our results showed that CD133 expression not only correlated with SAA expression, but also negatively correlated with Ki67 levels, indicating that CD133^+^ cells are quiescent (Fig. 2H, I). Last, in cell line phenotypic assay similar results were also observed, we found that SAA neutralization restricted the colony formation and wound healing ability (Fig. S2E–H), but have less effects on cell proliferation (Fig. S2B, C).

Here we reported that, SAA promotes stemness transformation in LUAD (primarily by promoting CSC self-renewal) and cell quiescence, which in turn promotes drug resistance. Furthermore, we observed that neutralizing antibodies against SAA can suppress these malignant phenotypes.

### SAA suppress α-PD-1 induced anti-tumor immunity in autologous ex-vivo organoids-PBMCs co-culture model

**Fig. 3:**
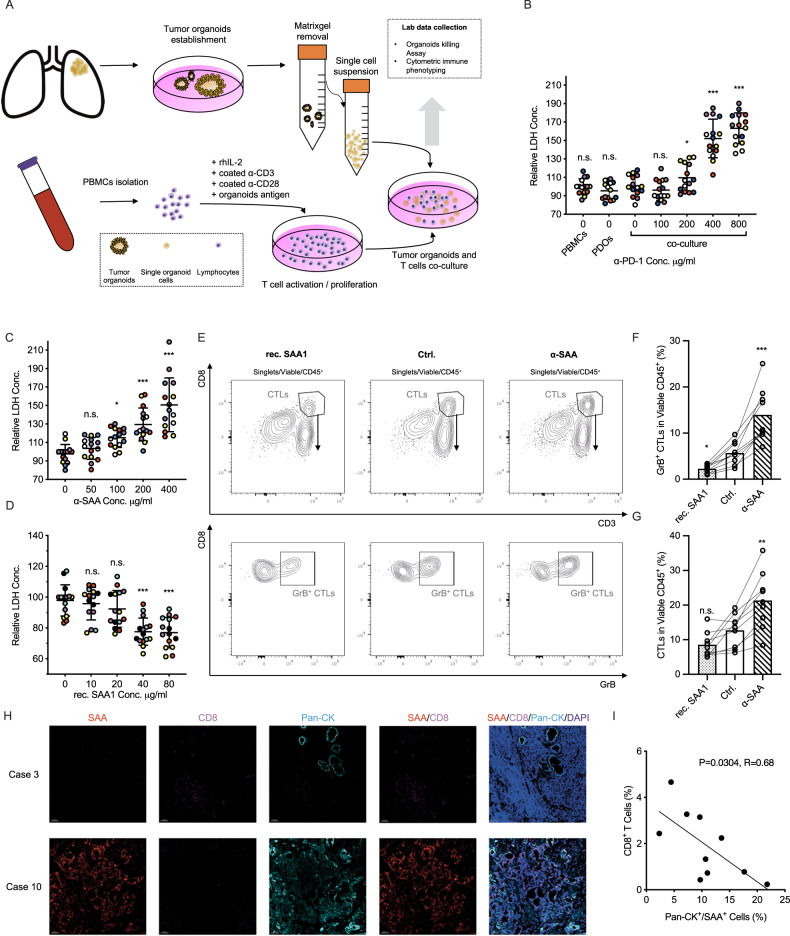
SAA suppresses α-PD-1 induced anti-tumor cytotoxic immune responses. **A** Flow chat of autologous ex-vivo tumor organoids-PBMCs co-culture model. **B** α-PD-1 (Tislelizumab) was introduced in the co-culture model and the supernatant were collected for LDH measurement (mean with SD, one way ANOVA with Post Hoc analysis). **C**, **D** The α-SAA neutralization antibody and rec. SAA1 protein were introduced in the co-culture model, after that the supernatant were collected for LDH measurement (immune-cytotoxic assay) (mean with SD, one way ANOVA with Post Hoc analysis). **E** α-SAA neutralization antibody and rec. SAA1 protein were introduced in the co-culture model, after 3 days cells were collected for immunophenotyping by flow cytometry. **F**, **G** the percentage of CD45^+^/CD3^+^/CD8^+^ CTLs and CD45^+^/CD3^+^/CD8^+^/GrB^+^ CTLs were measured (mean with SD, one way ANOVA with Post Hoc analysis). **H** Ten lung cancer FFPE samples were enrolled for immunofluorescence imaging, the SAA, CD8, Pan-CK and DAPI were stained. **I** The correlation between the percentage of CD8^+^ cells and SAA^+^ cells were evaluated (linear regression).

To explore the biological impact of SAA signal in the context of anti-tumor immunity, we established an autologous ex-vivo organoids-PBMCs co-culture model, as shown in Fig. 3A the fresh tumor and peripheral blood were collected from LUAD patients for tumor organoids culturing and PBMCs isolation, and the isolated PBMCs were first cryopreserved. After the successful establishment of tumor organoids, the PBMCs were first cultured with the exposure to tumor antigen (with the presence of α-PD-1, 0.2 mg/ml, Tislelizumab, BeiGene), for activation and expansion of T population, after that the PBMCs were co-cultured with prepared tumor organoids in 96 well U-bottom plate. After 72 h the co-cultured supernatant and cells were collected, the supernatant LDH level were measured for evaluating the immuno-cytotoxic efficiency and the cells were stained with several T cell markers for multiplex immunophenotyping by flow cytometry (Fig. S2B).

Prior to ex-vivo co-culture experiments, we performed bioinformatic analysis using the TCGA-LUAD dataset. We submitted the top 5 and bottom 5 SAA expression samples for GSEA analysis. We revealed a significant correlation between SAA expression and inflammatory response (Fig. S3B, C). Notably, we also observed a positive correlation between SAA expression and EMT as well as CD133 (Fig. S3A, B).

Tumor immune tolerance is a prevalent phenomenon in many types of cancer [25]. Clinically, α-PD-1 can block the interaction between the PD-1 receptor and its ligand PD-L1, which can shift the immune balance towards anti-tumor immunity. As we expected, tumor immune tolerance was also observed in the autologous organoids-PBMCs co-culture model. By measuring LDH level in the supernatant, we found that the level of cell death was similar in the PBMCs and organoids group compared with the co-culture group without α-PD-1 (Fig. 3B). Furthermore, we observed that 400 μg/ml α-PD-1 (Tislelizumab) was sufficient to induce significant immune-related cell death in the co-culture model (Fig. 3B).

Next, we introduced α-SAA and rec. SAA in an autologous organoids-PBMCs co-culture model using serial dilution (with 400 μg/ml Tislelizumab). We found that the rec. SAA significantly inhibited the killing effects in the co-culture model, while α-SAA promoted the killing effects (Fig. 3C, D, measured by LDH release in the supernatant). Additionally, we observed that SAA signal (40 μg/ml, rec. SAA1) notably hindered the expansion of CD45^+^/CD3^+^/CD8^+^ cytotoxic T lymphocytes (CTLs) and Granzyme B^+^ (GrB) CTLs. However, the use of SAA neutralization antibody (300 μg/ml) enhanced anti-tumor immunity and led to the expansion of CTLs and GrB+ CTLs populations (Fig. 3E–G). Finally, in 10 clinical lung cancer samples, we observed that the percentage of SAA^+^ cells was significantly negatively correlated with tumor-infiltrating CD8^+^ cytotoxic T lymphocytes (Fig. 3H, I).

In summary, we reported that the SAA has a potent biological impact on anti-tumor immunity, and that the α-SAA neutralization antibody significantly promotes the killing effects induced by α-PD-1 mediated anti-tumor immunity.

### SAA suppress anti-tumor immunity by driving T_H_2 polarization

Smole et al. reported that SAA is a soluble pattern recognition receptor that drives type 2 immunity [14]. However, anti-tumor immunity is primarily induced by T_H_1-mediated type 1 immunity responses [26]. Therefore, we further explored whether the SAA signal suppresses anti-tumor immunity by promoting the polarization of type 2 immunity.

In the organoids-PBMCs co-culture model, we investigated the impact of SAA on the releasing of cytokines and the polarization of T helper cells (T_H_s) using multiplex flow cytometry. We identified CD45^+^/CD3^+^/CD4^+^/IFN-γ^+^ T_H_s as T_H_1 cells and CD45^+^/CD3^+^/CD4^+^/IL-4^+^ T_H_s as T_H_2 cells (Fig. S2B), and the distribution of T_H_1 or T_H_2 cells reflects the balance between type 1 and type 2 immunity.

**Fig. 4:**
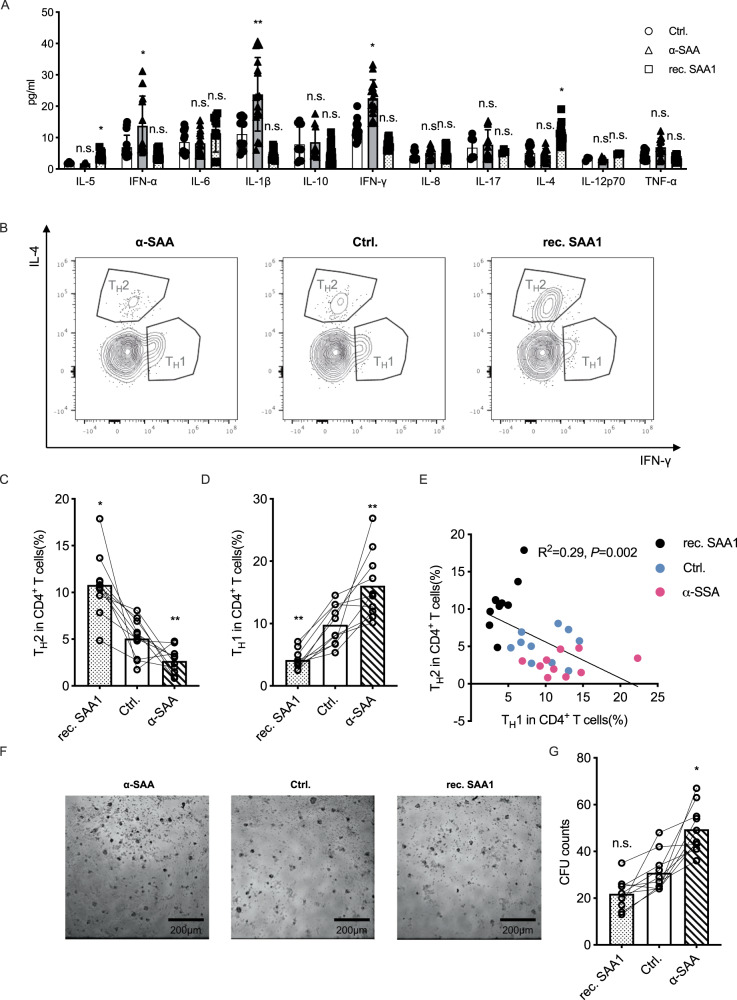
SAA suppress anti-tumor immunity by driving T_H_2 polarization. **A** α-SAA neutralization antibody and rec. SAA1 protein were introduced in the ex-vivo tumor organoids-PBMCs co-culture model (from 5 independent patients, separated data in Fig. S4A–E), after 3 days, the cells were collected for cytokine measurement using cytometric beads array. **B** α-SAA neutralization antibody and rec. SAA1 protein were introduced in the ex-vivo tumor organoids-PBMCs co-culture model, after 3 days, the cells were collected for immunophenotyping by flow cytometry, the CD45^+^/CD3^+^/CD4^+^/IFN-γ^+^ T_H_1 cells and CD45^+^/CD3^+^/CD4^+^/IL-2^+^ T_H_2 cells were measured. **C**, **D** the percentage of T_H_1 and T_H_2 cells were measured (mean with SD, one way ANOVA with Post Hoc analysis). **E** The co-relation between the percentage of T_H_1 and T_H_2 cells. **F**, additional co-culture experiments were performed in flat bottom 96 well plates (others in U-bottom plate). **G**, after 3 days the T cell clone formation unit (CFU) were counted in bright-field microscopy (mean with SD, one way ANOVA with Post Hoc analysis).

We found that the SAA signaling significantly influenced the releasing of many T_H_1 / T_H_2 polarization and anti-tumor immunity related cytokines including IFN-α, IL-1β, IFN-γ, IL-4 and etc. (Fig. 4A). Furthermore, we found that rec. SAA promotes the expansion of T_H_2 population while decrease the expansion of T_H_1 population, whereas α-SAA had the opposite impact on type 2 immunity polarization (Fig. 4B-D) and blocking IL-4 signal doesn’t influence the expansion of T_H_2 cells (Fig. S4F). The expansion of T_H_1 and T_H_2 cells are typically mutually exclusive, meaning that when one population is expanding, the other is suppressed. We also found a significant negative correlation between the expansion of T_H_1 and T_H_2 cells (Fig. 4E), indicating that the SAA signal has the ability to modulate the polarization of T helper cells and influence the balance between type 1 and type 2 immunity.

Furthermore, we observed that rec. SAA, the polarization towards type 2 immunity had a significant negative impact on anti-tumor immunity, as evidenced by a decrease in the number of colony formation units (CFUs) formed by T cells. In contrast, treatment with α-SAA led to a significant increase in the number of T cell CFUs formed (Fig. 4F, G).

In summary we found that SAA promotes the expansion of T_H_2 cells and suppresses the expansion of T_H_1 cells, leading to decreased anti-tumor immunity.

### SAA facilitate malignant cells release type II promoting cytokines

Type 2 immunity play a role in cancer initiation and progression, type 2 cytokines (e.g., IL-4 and IL-13) can inhibit the function of cytotoxic T cells, which limit the ability of T cell to eliminate cancer cells [15]. However, the relationship between type 2 immunity and cancer is complex and context-dependent, In the current study, we further evaluated the mechanism of SAA inducing type 2 immunity from the perspective of secretome.

**Fig. 5:**
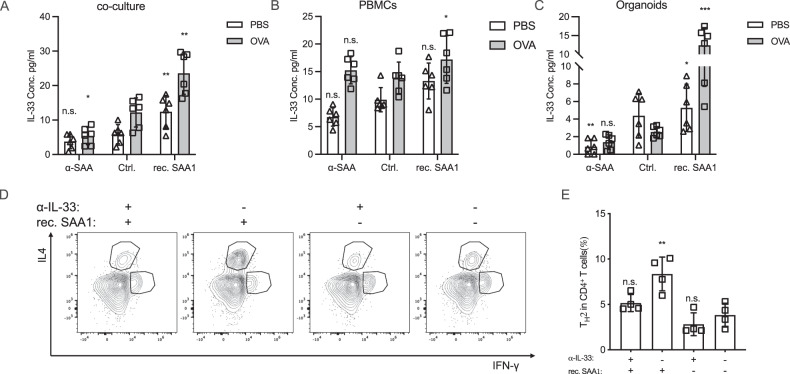
SAA drives malignant cells secrete type-2 immunity promoting cytokines. In the organoids-PBMCs co-culture, PBMCs or organoids culturing model, the OVA (Ovalbumin) were added to induce type 2 immunity. **A** After 3 days, the supernatant IL-33 concentration was measured in co-culture model (mean with SD, one way ANOVA with Post Hoc analysis). **B** After 3 days, the supernatant IL-33 concentration was measured in PBMCs culture model (mean with SD, one way ANOVA with Post Hoc analysis). **C** After 3 days, the supernatant IL-33 concentration was measured in organoids culture model (mean with SD, one way ANOVA with Post Hoc analysis). **D**, **E** The α-IL-33 neutralization antibody and rec. SAA1 protein was introduced in the co-culture model, after 3 days the cells were collected for immunophenotyping by flow cytometry (mean with SD, one way ANOVA with Post Hoc analysis).

OVA (ovalbumin) is a commonly used antigen to induce type 2 immune responses. In the co-culture model, it was found that OVA significantly induced the secretion of several type 2 inducing/promoting cytokines, including IL-33, IL-25, and TSLP, and rec. SAA further promoted the secretion of type 2 inducing/promoting cytokines, while α-SAA restricted the release of these cytokines (Fig. 5A and Fig. S5A and D). These findings suggest that SAA modulates the secretion of cytokines involved in type 2 immunity.

Next, to investigate which cell type exhibits the most significant changes in type 2 cytokine secretion in response to SAA signaling, we performed experiments using either malignant cells (organoids) or immunocytes (PBMCs) alone. It’s interesting to note that upon the stimulation or blockade of SAA signaling, organoids exhibit the most significant changes rather than the PBMCs in releasing type 2 cytokines, particularly IL-33 (Figs. 5B, C, S5B, C, E, and F), indicating that SAA and α-SAA are primarily modulating the organoids rather than directly affecting the immunocytes. In further exploration, an α-IL-33 neutralization antibody was introduced in the co-culture experiment. It was found that the α-IL-33 antibody was able to restrict the T_H_2 polarization (Fig. 5D, E) and promote the expansion of CTLs (Fig. S5G, H) induced by rec. SAA.

In summary, this indicates that the effect of SAA on promoting type 2 immunity is at least partially mediated by the induction of IL-33 secretion in malignant cells.

### SAA induced type 2 immunity promotes tumor fibrosis

Type 2 immunity and cytokines have been implicated in promoting tissue fibrosis, cytokines such as IL-4, IL-13, and TGF-β are predominantly produced by type 2 immune cells such as T_H_2 cells, which have been shown to play a key role in promoting fibrosis [15]. We further investigated the relationship between SAA signal and tumor fibrosis. The fibrocytes / fibroblasts were collected from primary LUAD samples based on their ability to adhere, while the suspended cells were collected for organoid culturing.

**Fig. 6:**
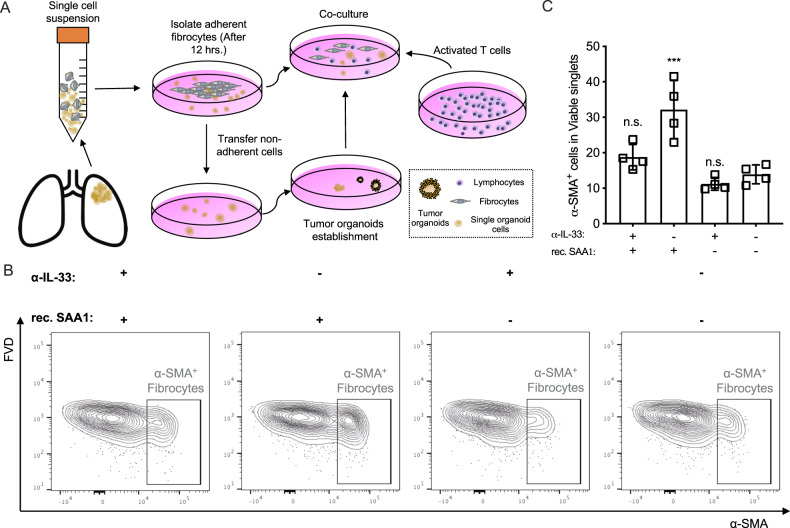
SAA induced secretome promotes fibrosis. **A** Flow chart of organoids-PBMCs-fibrocytes co-culture. **B**, **C** The α-IL-33 neutralization antibody and rec. SAA1 protein were introduced in the co-culture model, after 3 days the cells were collected for fibrocytes immunophenotyping by flow cytometry (α-SMA^+^ cells were identified as fibrocytes) (mean with SD, one way ANOVA with Post Hoc analysis).

The fibrocytes/fibroblasts from primary LUAD samples were collected and cryopreserved. Once the tumor organoids were established (normally after 15–25 days), a co-culture system was established with fibrocytes, PBMCs (prepared as described in Fig. 3A) and organoids to investigate the impact of SAA on tumor fibrosis (Fig. 6A).

In the fibrocytes-PBMCs-organoids co-culture system rec. SAA and α-IL-33 neutralization antibody were introduced, and the co-cultured cells were collected for immunophenotyping, α-SMA^+^ cells were identified as fibrocytes by flow cytometry. The results showed that the rec. SAA significantly promoted the expansion of fibrocytes, while blocking type 2 cytokines with α-IL-33 significantly restricted the SAA-induced expansion of fibrocytes (Fig. 6B, C). And α-SAA restricted the OVA induced tumor fibrosis (Fig. S6A). In summary, we found that, SAA induces type 2 immunity / cytokines promotes tumor fibrosis in lung adenocarcinoma.

### SAA-P2X7 interaction promotes cancer stem transformation and type 2 cytokine releasing

We further explored the mechanism underlying the phenotype that SAA promotes cancer stem transformation and the release of type 2 cytokines. It has been reported that SAA can act as a ligand for several receptors in different cell types / biological conditions, including TLR2/4 (Toll-like receptors 2/4) [27, 28], P2X7 (P2X purinoceptor 7) [29] and FPR2 (pairing formyl peptide receptor 2) [30]. But it’s unclear which receptor is dominating the SAA induced stemness transformation and type 2 immunity in the contest of lung cancer, thus we introduced siRNAs knockdown P2X7 and FPR2 (knockdown efficiency data in Fig. S6B) and TIRAP inhibitor (TIRAP is an adapter molecule associated with toll-like receptors) [31].

**Fig. 7:**
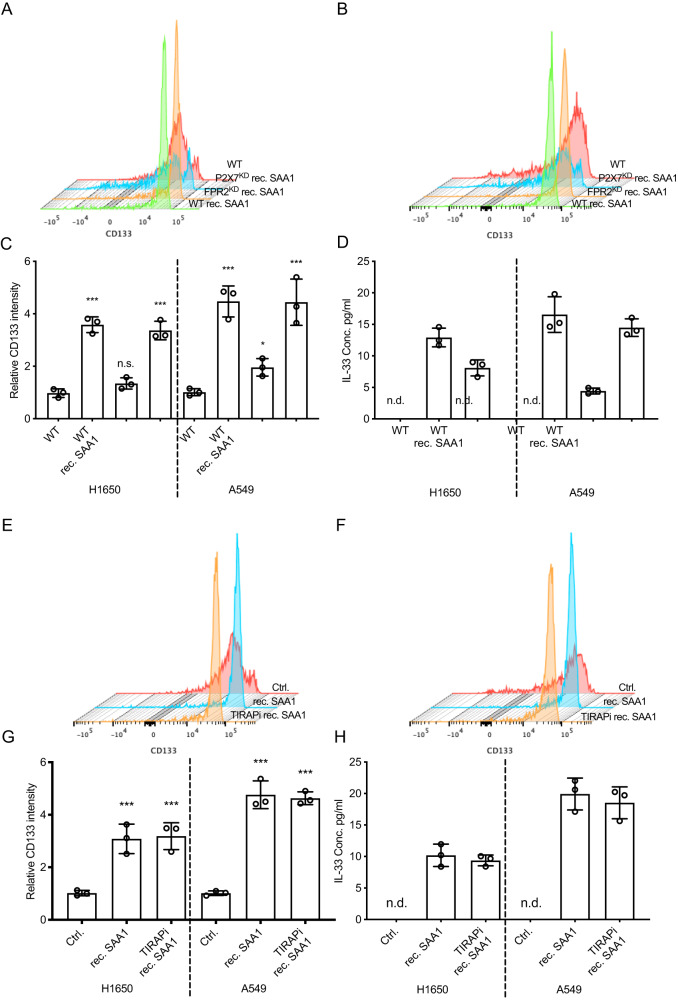
SAA-P2X7 interaction promotes cancer stem transformation and type 2 cytokine releasing. P2X7 or FPR2 knockdown and wildtype H1650 and A549 cell lines were treated with or without rec. SAA1. **A**–**C** The CD133 intensity on the cell surface were measured with flow cytometry (mean with SD, one way ANOVA with Post Hoc analysis). **D** The supernatant IL-33 were measured by ELISA (mean with SD, one way ANOVA with Post Hoc analysis). The wildtype H1650 and A549 cell lines were treated with or without TIRAPi (TIRAP inhibitor), **E**–**G** the CD133 were measured by flow cytometry (mean with SD, one way ANOVA with Post Hoc analysis). **H** The supernatant IL-33 were measured by ELISA (mean with SD, one way ANOVA with Post Hoc analysis).

Our findings indicate that knockdown of FPR2 in H1650 and A549 cell lines does not affect the elevated CD133 intensity or the release of IL-33 induced by SAA (Fig. 7A–D). Similarly, TIRAP inhibitor does not influence SAA-induced CD133 expression or IL-33 release (Fig. 7E–H). However, interestingly, knockdown of P2X7 significantly suppressed the cancer stem transformation and IL-33 release induced by SAA (Fig. 7A–D), suggesting the connection between SAA and P2X7.

In summary, our results suggest that in lung cancer, the cancer stemness transformation and release of type 2 immunity induced by SAA is dependent on P2X7.

## Discussion

It has been reported that cancer stem cells represent a subpopulation of cancer cells capable of self-renewal, tumor initiation, and therapeutic resistance [5]. In our previous study, we reported that the cancer stem cell population is highly resistant to chemotherapy due to its quiescence behavior [8]. In current study, we used scRNA-Seq technology to explore intratumor heterogeneity in solid tumors. Single-cell sequencing (scRNA) technologies enable unbiased high-throughput analysis with minimal sample input volumes and provide a way to study the functional state of individual cells [32, 33].

The relationship between cancer stem population and inflammation is reported to be complex and multifaceted, with both factors potentially influencing each other in a bidirectional manner [34, 35]. Our findings suggest that in addition to their self-renewal, tumor initiation, and therapeutic resistance properties, CSCs are also highly inflammatory and secrete many inflammatory factors in the tumor microenvironment. Next, we found that, in the tumor microenvironment the acute phase protein SAA was preliminary secreted by CD133^+^ cancer stem population.

SAA is a family of small, acute-phase proteins that are produced primarily in response to inflammation and tissue injury [9]. Elevated levels of SAA have been observed in various types of cancer [10, 24, 36]. However, the reason why cancer cells produce high amounts of SAA is still unclear. We first investigated the impact of SAA on cancer stem transformation. In tumor organoids, we show that SAA promotes the expansion of the cancer stem cell population. Additionally, we found that recombinant SAA significantly increases drug resistance by inducing cancer stem cell transformation. Importantly, we also found that neutralizing SAA with an antibody improves chemosensitivity (Fig. 2). This suggests that α-SAA neutralization antibody may have therapeutic potential in chemotherapy. Studies have shown that SAA may play a role in cancer development and progression by promoting, and metastasis. SAA can also modulate the immune response, potentially leading to tumor evasion and resistance to therapy [37, 38].

As SAA is a secreted acute phase protein, we also investigated its impact on anti-tumor immunity. To better understand the host anti-tumor microenvironment in human, we designed and performed an autologous ex-vivo organoid-PBMC co-culture model. This model allows us to measure the immune-cytotoxic effect of T cells on autologous malignant organoids and explore the differentiation characteristics of T cells. Our findings suggest that the SAA signal restricts the polarization of type 1 immunity while promoting polarization towards type 2, This occurs through the regulation of malignant cells secrete type 2 cytokines such as IL-33. Ultimately, this limits the cytotoxic killing effects of T cells to malignant cells, which is mediated by the PD-1 antibody in our ex-vivo co-culture model (Figs. 3, 4 and 5).

Interestingly, Smole et al. also reported that SAA acts as a soluble pattern recognition receptor that drives type 2 immunity [14]. They found that SAA drives the pulmonary epithelial release type 2-promoting cytokines, such as IL-33, in a SAA1-dependent manner. These results are consistent with our findings and suggest that malignant lung cancer cells could exploit this physiological allergenic mechanism to promote the polarization of type 2 immunity and restrict anti-tumor killing.

Type 2 cytokines, such as IL-4, IL-5, and IL-13, are also known to be involved in the activation and differentiation of fibroblasts [15]. Moreover, type 2 cytokines can also stimulate the recruitment and activation of other cells involved in the fibrotic process [39, 40]. For example, IL-4 and IL-13 can induce the polarization of macrophages towards an alternatively activated phenotype, which is associated with tissue repair and fibrosis [41]. Tumor fibrosis is commonly triggered by chronic inflammation dominated by type 2 cytokines, which are often present in the tumor microenvironment [42], and tumor fibrosis has been associated with poor prognosis and increased malignancy in several types of cancer [43, 44]. We showed that SAA induced type 2 immunity significantly promoted the expansion of fibrocytes, indicating its involvement in tumor fibrosis. Moreover, the use of α-SAA neutralization antibody led to a significant reduction in OVA induced tumor fibrosis. For mechanism exploration, although SAA has been reported have many receptors, but we found that in the context of lung cancer, the SAA induced lung cancer cell stemness transformation is dependent on P2X7.

Thus, our research identified lung cancer stem population secreted acute phase protein SAA promotes cancer stemness transformation, tumor fibrosis and restricts anti-tumor immunity by driving type 2 immunity and dependent on SAA-P2X7 interaction, notably we also highlighted the therapeutic potential of α-SAA neutralization antibody.

## Materials and methods

### Cell line and transfection, primary sample preparation, and ex-vivo experiments

H1650 cells and A549 cells were obtained from the American Type Culture Collection (ATCC, USA) and no mycoplasma contamination was observed. Cells were cultured in RPMI1640 medium (Thermo Fisher) supplemented with 10% fetal bovine serum and 1% penicillin / streptomycin (Thermo Fisher) and cultured at 37 °C in a humidified incubator containing 5% CO_2_, and for cell line transfection Lipofectamine 3000 were used following official instruction with following siRNA, P2X7: 5′-CUUUAACGUCGGCUUGGGCUC-3′ and 5′-GCCCAAGCCGACGUUAAAGUA-3′, FPR2: 5'-GGAGAUGUGUAUUGUACAUTT-3' and 5'-AUGUACAAUACACAUCUCCTT-3'.

Fresh tumor resections were collected and immediately placed on ice after surgical removal from patients with lung adenocarcinoma (LUAD). The samples were stored in sterile tubes containing primary sample stocking buffer (#PDO-0012, Nanjing Faun Biological and Technology Co. Ltd.) and transported to the laboratory on ice. The samples were transported to the lab within 24 h, to ensure optimal quality. Upon arrival the tumor tissues were washed with primary sample washing buffer (#PDO-0013, Nanjing Faun Biological and Technology Co. Ltd.) for 30 min at room temperature on an orbital shaker. After that the tumor tissue was diced into small pieces (approximately the size of a sesame seed), and digested using enzymatic digestion buffer (#PDO-0016, Nanjing Faun Biological and Technology Co. Ltd.) in a thermoshaker at 37 °C for 30–50 min. And the cell suspensions were vigorously pipetted to ensure complete dissociation and passed through a 70 μm cell strainer. The cell suspensions were then resuspended in Matrixgel (40183ES10, Ceturegel® Yeasen Biotechnology) and seeded in 6-well plates. For the first 48 h, primary cells were cultured in lung cancer organoids culture medium (#PDO-L006, Nanjing Faun Biological and Technology Co. Ltd.) with ROCKi (10 µM, ROCK inhibitor, only for the first 24 h!) [45, 46], and the fresh medium without ROCK inhibitor was changed every 48 h.

Organoids should be split when confluent or when their diameter exceeds 300 μm. Established organoids can be passaged every 15–25 days at a 1:2–1:4 ratio. To begin the passaging process, the complete organoid medium should be removed, then organoid-Matrixgel drops should be resuspended in organoids passaging buffer (#PDO-0016, Nanjing Faun Biological and Technology Co. Ltd. 1 mL per well) and incubated at 37 °C for 5–15 min, after that the suspension should be resuspended with pipettes every 5 min. To inhibit digestion, the mix should be diluted 10-fold with cold PBS. The mixture should then be spun at 300 g for 5 min at 4 °C, and remove supernatant. This step should be repeated at least 2 times for fully gel removal.

For tumor reactive T cell preparation, collect 5 ml whole blood in EDTA-containing tubes. Dilute the blood 1:1 with PBS in a 50 ml tube. Layer the diluted blood over 15 mL of Ficoll-Paque Plus (Cytiva) in conical tube. Centrifuge the tube at 400 g for 30 min at room temperature with no brake. Collect the interface layer containing the PBMCs and transfer it to a new 50 mL conical tube. Wash the PBMCs by adding PBS to the tube until it is nearly full. Centrifuge at 300 g for 10 min at room temperature with a brake. Discard the supernatant and resuspend the cell pellet in PBS or the appropriate buffer for downstream applications. For tumor reactive T cell preparation, the PBMCs were cultured with plate bond functional grade CD3 / CD28 antibody (#TC0011, Nanjing Faun Biological and Technology Co. Ltd.), and supplement with IL2 (#TC0035, Nanjing Faun Biological and Technology Co. Ltd.), PD-1 antibody (Tislelizumab, BeiGene) and tumor antigen (organoids treated with 50 Gy Ionizing radiation) for 3 days. For PDOs-PBMCs co-culture, the above tumor organoids and tumor reactive T cells were collected and counted, and immunocytes-organoid cells were co-cultured at the ratio of 5:1 to 8:1 at U-bottom 96 well plates (some case with flat bottom 96 well plates), after 3 days the co-cultured cells and supernatant were collected for further analysis. The antibody or protein introduced in the co-culture model include SAA antibody (# 924903, R&D), IL-33 antibody (# 40015D, R&D), rec. human SAA1 (300-53, PeproTech) and IL-4R neutralization antibody (10402-R401, SinoBiological).

For cell line phenotypic assay, The Cell Counting Kit-8 (C0038, Beyotime) was used to determine the cell proliferation rate, 2000 cells/well were seeded in 96-well plates and the absorbance was measured using a plate reader. In wound healing assay, cells were seeded in six-well plates, after 24 h an artificial scratch wound on a confluent monolayer of cells was created, then serum-free medium was added, cells were imaged at baseline and 24 h. For colony formation assays, a total of 200 cells were placed in a fresh 6-well plate and maintained in media containing 10% FBS, after 10 days, cells were fixed with 4% paraformaldehyde and stained with 0.1% crystal violet. Visible colonies were manually counted.

### Flow cytometry analysis and supernatant soluble protein / enzyme activity measurement

For FACS staining, cells were washed and resuspended at the concentration of 10^6^ cells/ml in PBS for Fixable Viability Dye (FVD) staining (Thermo fisher, 65-0866-14, 65-0867-14, 65-0863-14 or L34957) at 15 min in room temperature. Then cells were pre-incubated with blocking buffer (#FH0008, Nanjing Faun Biological and Technology Co. Ltd.) for 15 min and subsequently stained with FACS antibodies (in Table S1) for surface staining at 4 degree 20 min, then cells were fixed and permeabilized (IntraStain kit, F0063, Nanjing Faun Biological and Technology Co. Ltd.). After that cells were intracellular stained at room temperature for 20 min (antibodies in Table S1). For cell cycle distribution assay, cells were harvested and fixed, after that cells were washed with PBS twice followed by Hoechst 33342 (Sigma), Pyronin y (Sigma) and Pan-CK staining. Flow cytometry analysis and cell sorting were performed on a FACSAria II flow or a FACSCelesta cytometer (BD Biosciences). Data were analyzed by FlowJo (version 10.4; FlowJo LLC). Each assay was performed in triplicate.

For supernatant cytokine measurement the following ELISA kits were used, IL-33, 900-M398, PeproTech; IL-25, 900-M234, PeproTech; TSLP, 88-7497-22, Thermo Fisher. And the data were collected in a 96 well plate reader that measure optical density (O.D.) at 450 nm. And for supernatant LDH measurement, the C0016 from Beyotime was used. For tagging cells, following reagent were introduced from Thermo Fisher, CellTracker, C10094 and C2927. For other inflammatory cytokine measurement in Fig. 4A and Fig. S4A–E, the BD clinical IVD level cytometric beads array was used in department of clinical laboratory in our hospital.

### Single-cell sequencing analysis

To investigate the biological characteristics of SAA and PROM1 (CD133), we analyzed scRNA-Seq tumor data (GSE131907) [22]. We evaluated the number of RNA features, absolute UMI numbers, and mitochondrial gene distribution per sample, and established the criteria for selecting samples with nFeature ≥2000, 2000 ≤ nCount ≤ 5000, and percent.mt ≥5%. To further reduce confounding factors, we included the ratio of red blood cells, as they lack a nucleus and a transcriptome. Using this feature, we calculated the ratio of red blood cells.

We then applied the T-distributed stochastic neighborhood embedding (t-SNE) algorithm to visualize the cell relationships and counted the genes for each cluster. Using the “SingleR” R package [47], we identified the cell types in the dataset. Epithelial cell populations were defined and subjected to copy number variation analysis using the “infercnv“ R package [48, 49]. Finally, we visualized the expression differences of SAA and PROM1 in different samples.

### Immunofluorescence imaging and Western blot

For immunofluorescence imaging, a total of ten FFPE (Formalin-Fixed Paraffin-Embedded) samples from lung cancer patients were retrospectively enrolled, the paraffin sections (5 µm) of samples were used and processed for immunofluorescence staining. Paraffin sections were deparaffinized by heating for 1 h at 60 °C, then rehydrated twice in Xylene for 5 min, Antigen retrieval was performed with an unmasking solution, followed by blocking, and incubation with direct Immunofluorescence primary antibody (overnight, 4 °C), SAA-APC; CD133-AF488; Pan-CK-AF594; Ki67-BV605 and CD8-AF488 (details in Table S1) followed with DAPI (C1005, Beyotime) staining and mounting.

Images were taken by Dragonfly 500 confocal microscope system (Andor, Oxford Instruments). For quantification, five different fields were imaged from different experiments and the indicated mean fluorescence intensity was calculated by Imaris 9.9.0 software (Oxford Instruments). All the images were acquired at the same exposure time, magnification and fluorescence intensity.

The cells were lysed using RIPA lysis buffer, and the protein concentration was determined using a BCA protein assay kit (Beyotime, China). The protein was then electrophoresed on a 10% SDS-PAGE gel and transferred onto a polyvinylidene difluoride (PVDF) membrane. After blocking, the membranes were incubated overnight at 4 °C with primary antibodies (details in antibody list), followed by incubation with a horseradish peroxidase (HRP)-conjugated secondary antibody for 1.5 h at room temperature. Finally, the enhanced chemiluminescence (ECL) kit (Beyotime, China) was used to detect blots.

### Statistical analysis

The gene set enrichment analysis (GSEA) was performed following official instruction. Data were presented as the mean ± SD (standard deviation) and analyzed using a student’s *t*-test. *P* < 0.05 was considered to indicate a statistically significant difference (*P < 0.05, **P < 0.005, ***P < 0.0005). Excel (Office 365, Microsoft) and GraphPad Prism (version 9.5.1, GraphPad Software, Inc) software were used for statistical analyses and the production of graphs.

## Availability of data and materials

All the datasets used in the paper are cited with Gene Expression Omnibus Accession Number. All data generated and analyzed during this study are included in this published article and its Supplementary Information files. Additional data are available from the corresponding author on reasonable request.

### Supplementary information

SUPPLEMENTAL MATERIAL

Original Data File

aj-checklist

## Data Availability

All the datasets used in the paper are cited with Gene Expression Omnibus Accession Number. All data generated and analyzed during this study are included in this published article and its  Supplementary Information files. Additional data are available from the corresponding author on reasonable request.

## Materials availability

All the datasets used in the paper are cited with Gene Expression Omnibus Accession Number. All data generated and analyzed during this study are included in this published article and its Supplementary Information files. Additional data are available from the corresponding author on reasonable request.
