# Supplementary material for: SAA suppresses α-PD-1 induced anti-tumor immunity by driving TH2 polarization in lung adenocarcinoma
Source: Cell Death Dis. 2023 Nov 4;14(11):718. doi: 10.1038/s41419-023-06198-w (PMC10625560; doi:10.1038/s41419-023-06198-w)
Supplement: Supplementary file 1 — SUPPLEMENTAL MATERIAL [file 41419_2023_6198_MOESM1_ESM.pdf]

SAA suppresses α-PD-1 induced anti-tumor immunity by driving T_H2 polarization in lung adenocarcinoma

Xin Wang[1,9], Shaodi Wen[1,9], Xiaoyue Du[1], Yihan Zhang[1], Xiao Yang[2], Renrui Zou[1], Bing Feng[1], Xiao Fu[3, 4], Feng Jiang[5], Guoren Zhou[1], Zi Liu[6], Wei Zhu[7], Rong Ma[8], Jifeng Feng[1, #], Bo Shen[1, #]

1, Department of Oncology, The Affiliated Cancer Hospital of Nanjing Medical University, Jiangsu Cancer Hospital, Jiangsu Institute of Cancer Research, Nanjing, China.
2, Department of clinical Laboratory, Jiangsu Cancer Hospital, The Affiliated Cancer Hospital of Nanjing Medical University, Jiangsu Institute of Cancer Research, Nanjing, China.
3, Department of General Surgery, Nanjing Drum Tower Hospital
4, Clinical College of Traditional Chinese and Western Medicine, Nanjing University of Chinese Medicine, Nanjing, China
5, Department of Thoracic Surgery, Jiangsu Cancer Hospital, The Affiliated Cancer Hospital of Nanjing Medical University, Jiangsu Institute of Cancer Research, Nanjing, China.
6, Nanjing Advanced Analysis Tech. (NAAT) Co., LTD.
7, School of Medicine, Jiangsu University, Zhenjiang, China.
8, Research Center for Clinical Oncology, Jiangsu Cancer Hospital & Jiangsu Institute of Cancer Research & The Affiliated Cancer Hospital of Nanjing Medical University
9, These authors contributed equally to this work: Xin Wang & Shaodi Wen

Correspondence[#],

Jifeng feng, Department of Oncology, The Affiliated Cancer Hospital of Nanjing Medical University, Jiangsu Cancer Hospital, Jiangsu Institute of Cancer Research, Nanjing, China. Email: fjif@jszlyy.com.cn

Bo Shen, Department of Oncology, The Affiliated Cancer Hospital of Nanjing Medical University, Jiangsu Cancer Hospital, Jiangsu Institute of Cancer Research, Nanjing, China. Email: shenbo987@njmu.edu.cn

Supplement Figure 1-1,

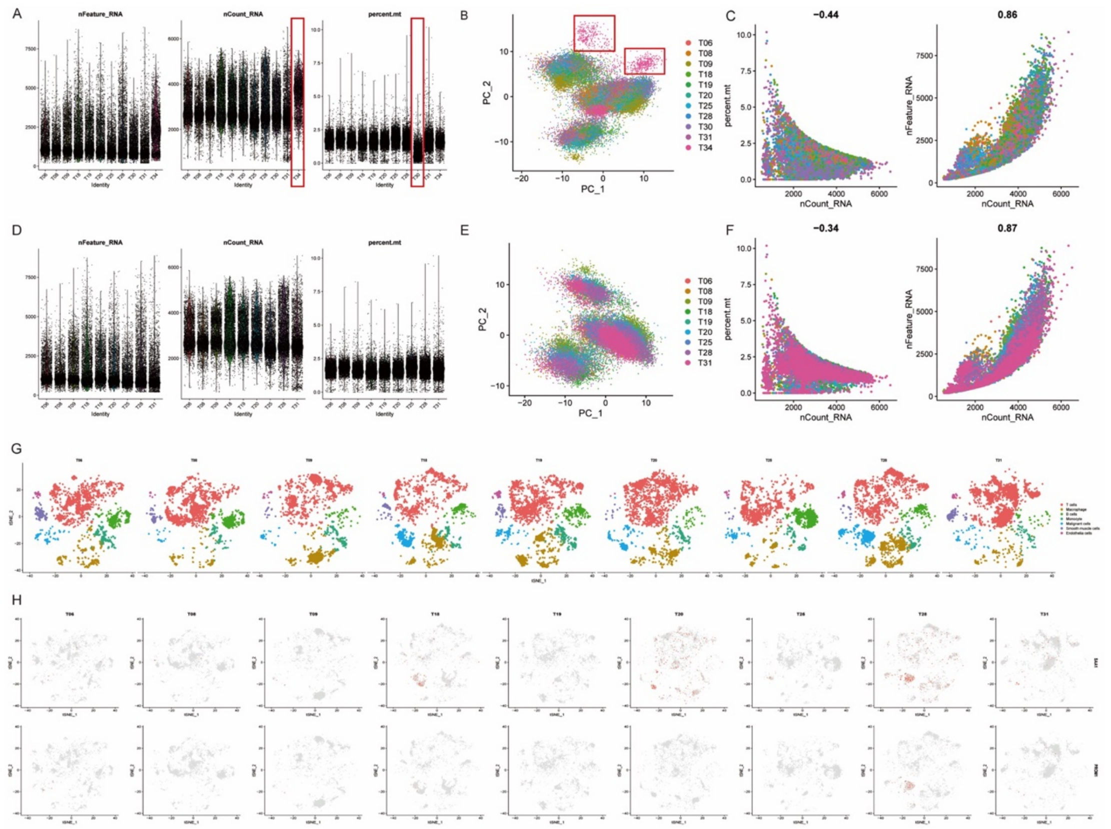

A. The RNA signature numbers, absolute UMI numbers, and mitochondrial genes of 11 lung adenocarcinoma samples. B, to further explore the variation in the samples, a principal component analysis (PCA) plot was generated. The red markers on the plot indicate outlier PCA cells, emphasizing the importance of quality control measures in our analysis. C, the correlation between the sum of expression levels of all genes in the cell, the number of genes whose expression levels are greater than 0 (nFeature_RNA), and the percentage of mitochondrial gene expression levels (percent.mt) detected in this cell. D, after quality control, 9 primary lung adenocarcinoma samples were identified. E, A PCA map was also generated for 9 samples. F, The correlation between nCount, nFeature_RNA, and percent.mt in the 9 lung adenocarcinoma samples. G, The distribution of cells across different samples. H，the expression of SAA1 and PROM1 in different samples.

Supplement Figure 1-2,

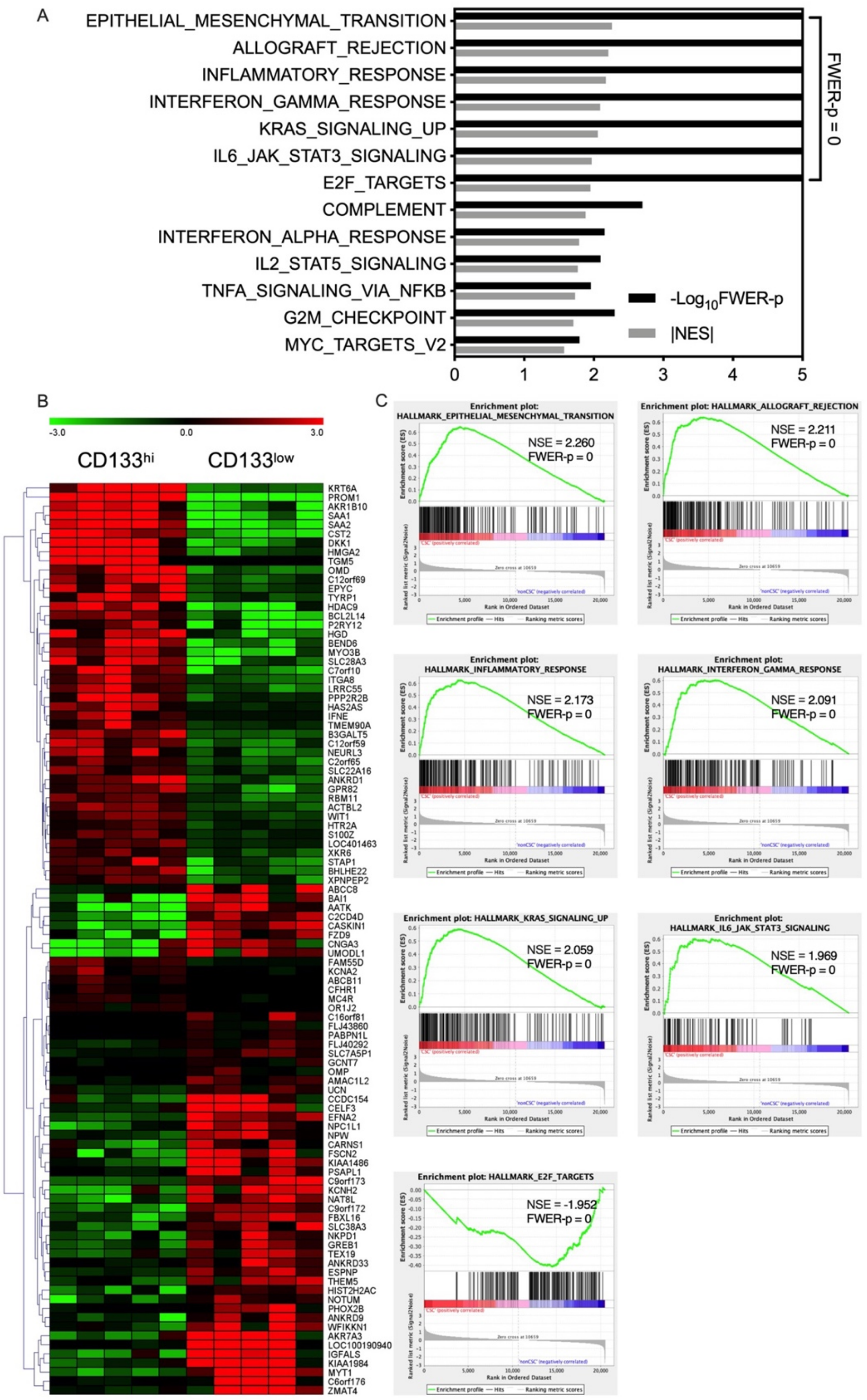

A, B & C, The TCGA (The Cancer Genome Atlas) database was utilized to conduct a comprehensive gene set enrichment analysis (GSEA) on LUAD samples, the top 5 and bottom 5 CD133 expression samples for GSEA analysis.

Supplement Figure 2,

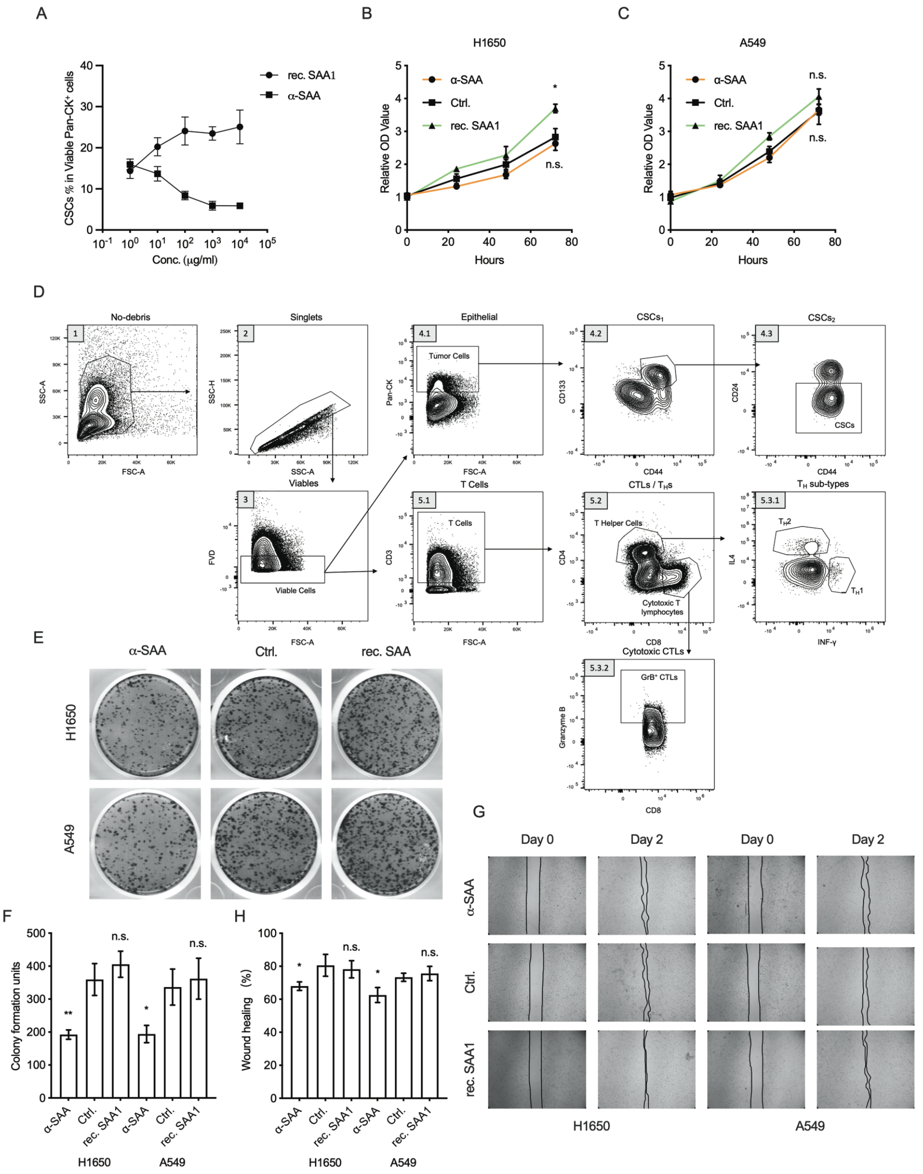

A, the best effecting concentration of α-SAA and rec. SAA were determined by measuring their ability to induce or suppress stemness transformation in organoid sample in a serial dilution assay. B & C, CCK8 assay was used to evaluate the cell proliferation rate. D, The gating strategy for immunophenotyping of cell samples from tumor organoids-PBMCs co-culture model by flow cytometry. E & F, the colony formation assay was used for evaluating the colony formation ability. G & H, the wound healing assay was introduced for evaluating the migration ability.

Supplement Figure 3,

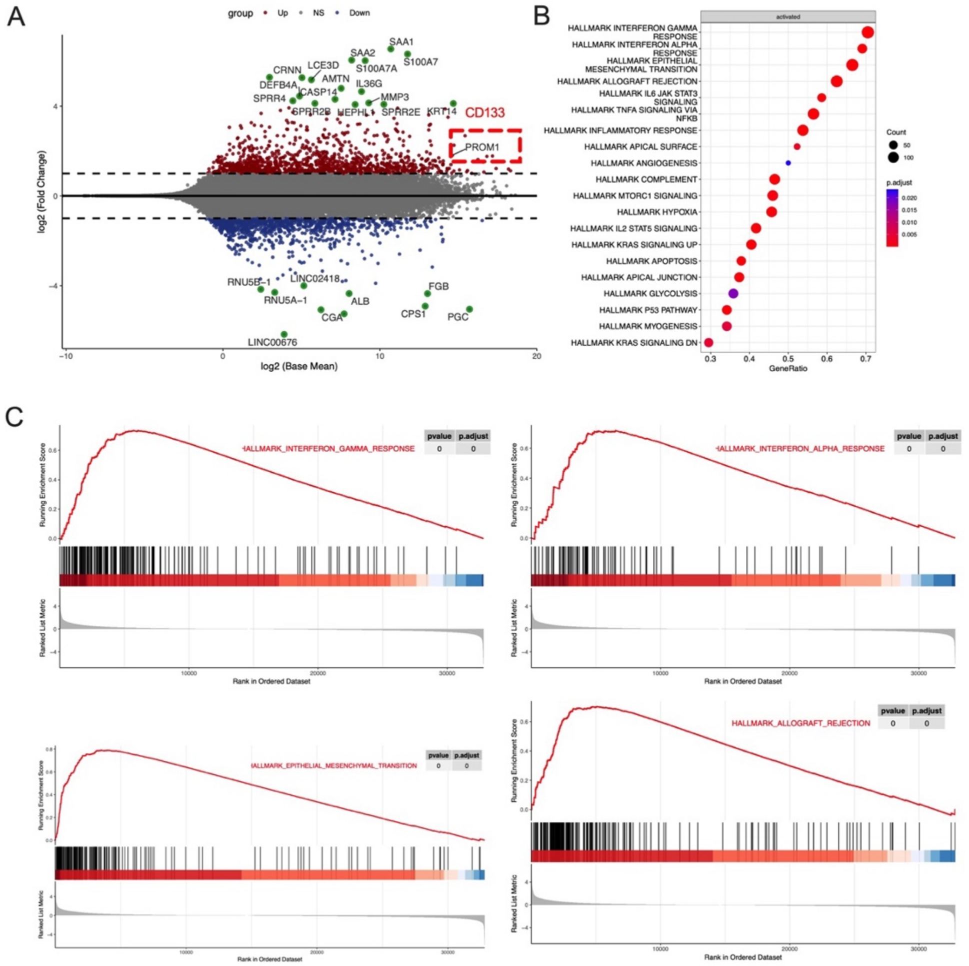

A, B & C, The TCGA (The Cancer Genome Atlas) database was utilized to conduct a comprehensive gene set enrichment analysis (GSEA) on LUAD samples, the top 5 and bottom 5 SAA1 expression samples for GSEA analysis.

Supplement Figure 4,

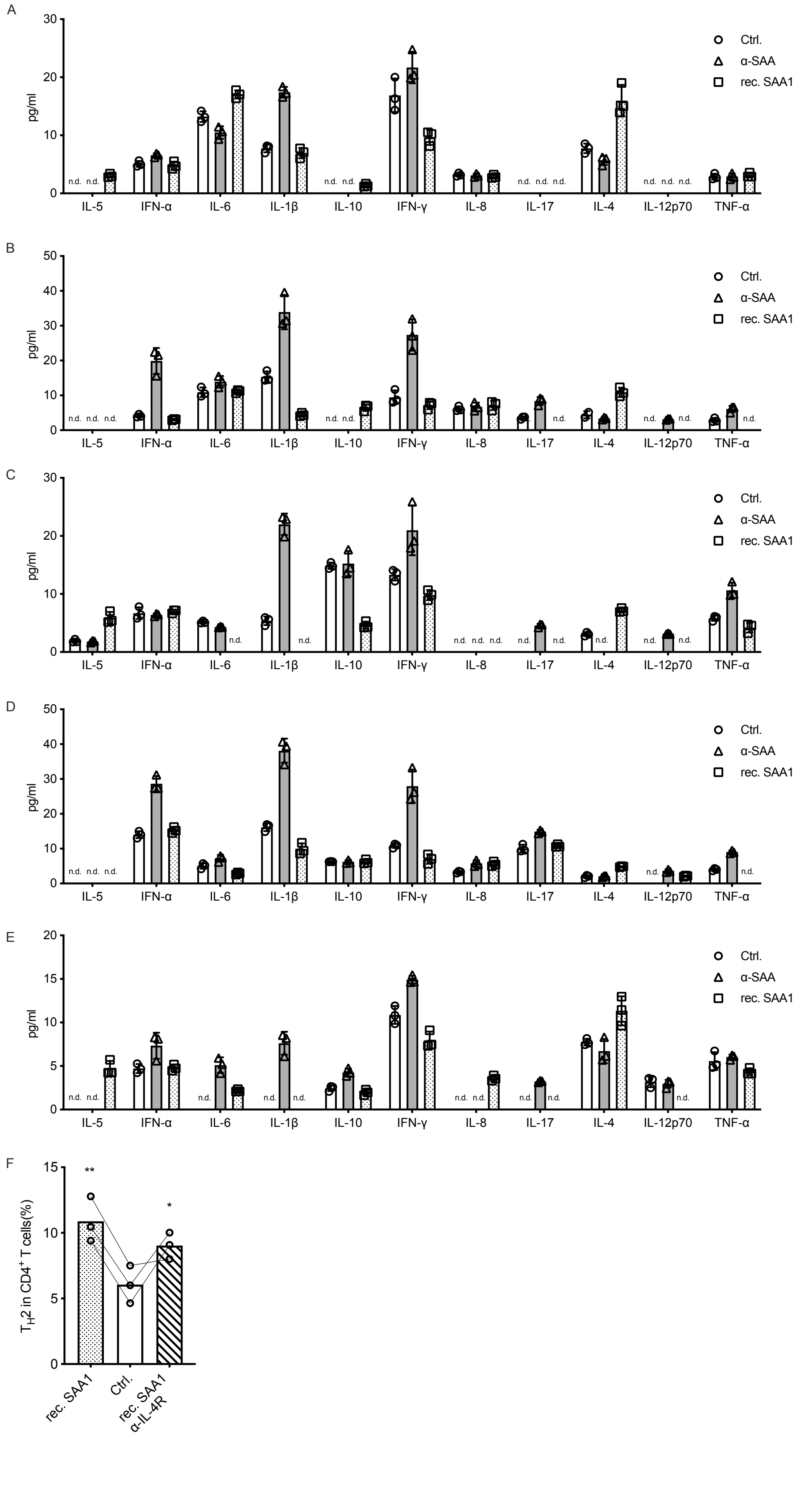

A-E, the cytokine measurement in 5 independent co-culture experiment by cytometric beads array (related with Fig. 4A).

F, the α-IL-4R neutralization antibody and rec. SAA were introduced in the co-culture model, after 3 days the cells were collected for immunophenotyping by flow cytometry measuring the percentage of T_H2 cells.

Supplement Figure 5,

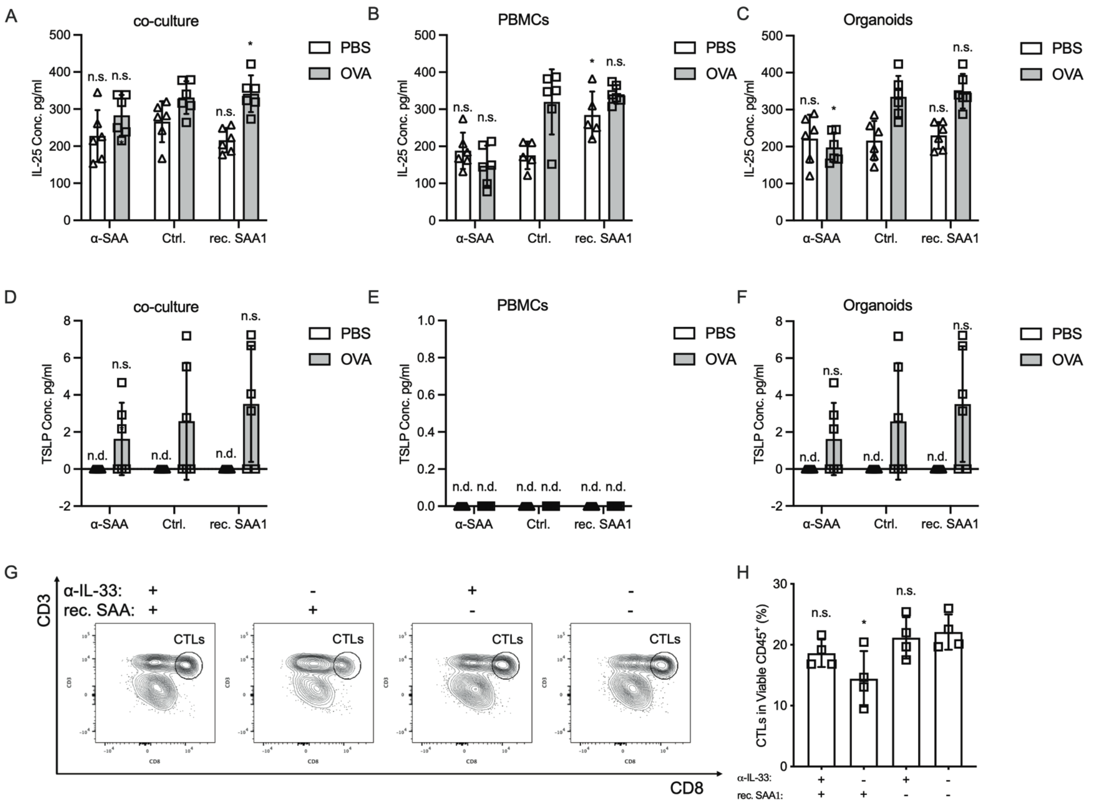

In the organoids-PBMCs co-culture, PBMCs or organoids culturing model, the OVA (Ovalbumin) were added to induce type 2 immunity. After 3 days, A & D, the supernatant IL-25 and TSLP concentration were measured in co-culture model. B & E, the supernatant IL-25 and TSLP concentration were measured in PBMCs culture model. C & F, the supernatant IL-25 and TSLP concentration were measured in organoids culture model. G & H, the α-IL-33 neutralization antibody and rec. SAA1 protein was introduced in the co-culture model, after 3 days the cells were collected for immunophenotyping by flow cytometry exploring the percentage of CTLs.

Supplement Figure 6,

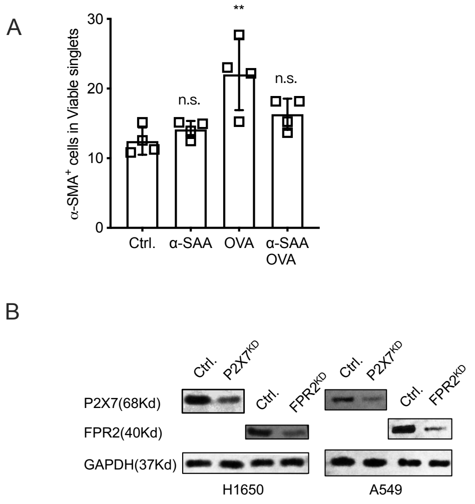

A, the α-SAA neutralization antibody and OVA was introduced in the organoids-PBMCs-fibrocytes co-culture model, after 3 days the cells were collected for fibrocytes immunophenotyping by flow cytometry (α-SMA^{+} cells were identified as fibrocytes). B, the knockdown efficiency of P2X7 and FPR2 in A549 and H1650 cells.

Table S1, antibody list

**Antibody**	**Clone**	**Vender**	**Application**
CD3	SK7	NAAT	Flow Cytometry
CD4	SK3	NAAT	Flow Cytometry
CD8	SK1	NAAT	Flow Cytometry
IL4	11B11	NAAT	Flow Cytometry
IFN-γ	XMG1.2	NAAT	Flow Cytometry
GrB	GB11	NAAT	Flow Cytometry
Pan-CK	AE1/AE3	NAAT	Flow Cytometry
CD45	2D1	NAAT	Flow Cytometry
CD44	IM7	Thermo Fisher	Flow Cytometry
CD133	EMK08	Thermo Fisher	Flow Cytometry
CD24	ML5	BioLegend	Flow Cytometry
CD326	MH99	Thermo Fisher	Flow Cytometry
SAA	Polyclonal (bs-19359R-APC)	Bioss	Immunofluorescence imaging
CD133	EPR20980-104	Abcam	Immunofluorescence imaging
Pan-CK	C-11	Abcam	Immunofluorescence imaging
Ki67	Ki-67	Biolegend	Immunofluorescence imaging
CD8	EPR21769	Abcam	Immunofluorescence imaging
PD-1	NA(Tislelizumab)	BeiGene	functional grade
SAA	924903	R&D	functional grade
CD3	OKT3	NAAT	functional grade
CD28	CD28.2	NAAT	functional grade
α-SMA	1A4	Thermo Fisher	Flow Cytometry
FPR2	Polyclonal (ABF118)	Sigma	Western Blot
P2X7	D-1	Santa Cruz	Western Blot
GAPDH	1E6D9	Proteintech	Western Blot
