# Supplementary figures and images for: SAA suppresses α-PD-1 induced anti-tumor immunity by driving TH2 polarization in lung adenocarcinoma
Source: Cell Death Dis. 2023 Nov 4;14(11):718. doi: 10.1038/s41419-023-06198-w (PMC10625560; doi:10.1038/s41419-023-06198-w)

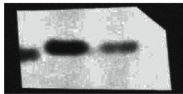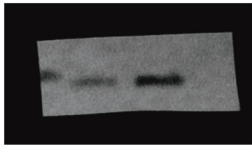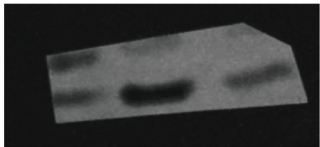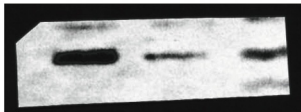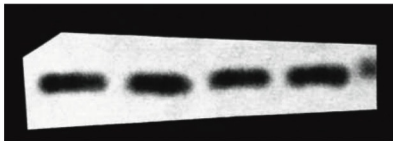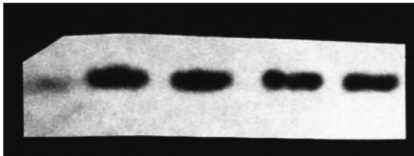

Supplement: Supplementary file 2 — Original Data File [file 41419_2023_6198_MOESM2_ESM.pdf]
